# Supplementary material for: Multi‐dataset identification of innovative feature genes and molecular mechanisms in keratoconus
Source: J Cell Mol Med. 2024 Sep 19;28(18):e70079. doi: 10.1111/jcmm.70079 (PMC11412914; doi:10.1111/jcmm.70079)
Supplement: Supplementary file 2 — Material S2. [file JCMM-28-e70079-s002.zip › 7.GSEA analysis/5.RNF217-AS1/KEGG_NATURAL_KILLER_CELL_MEDIATED_CYTOTOXICITY.html]

Details for gene set KEGG\_NATURAL\_KILLER\_CELL\_MEDIATED\_CYTOTOXICITY[GSEA]

|  || Dataset | RNF217-AS1.group.cls#h\_versus\_l |
| Phenotype | group.cls#h\_versus\_l |
| Upregulated in class | h |
| GeneSet | KEGG\_NATURAL\_KILLER\_CELL\_MEDIATED\_CYTOTOXICITY |
| Enrichment Score (ES) | 0.19892216 |
| Normalized Enrichment Score (NES) | 0.7392734 |
| Nominal p-value | 0.95303327 |
| FDR q-value | 1.0 |
| FWER p-Value | 1.0 |
Table: GSEA Results Summary

  

Fig 1: Enrichment plot: KEGG\_NATURAL\_KILLER\_CELL\_MEDIATED\_CYTOTOXICITY      
 Profile of the Running ES Score & Positions of GeneSet Members on the Rank Ordered List

  

| SYMBOL | TITLE | RANK IN GENE LIST | RANK METRIC SCORE | RUNNING ES | CORE ENRICHMENT || 1 | SH3BP2 | na | 152 | 0.688 | 0.0332 | Yes |
| 2 | PRKCG | na | 203 | 0.657 | 0.0676 | Yes |
| 3 | NFATC2 | na | 219 | 0.642 | 0.1022 | Yes |
| 4 | IFNA14 | na | 927 | 0.451 | 0.1064 | Yes |
| 5 | PIK3R5 | na | 1058 | 0.434 | 0.1264 | Yes |
| 6 | IFNA4 | na | 1517 | 0.391 | 0.1345 | Yes |
| 7 | PPP3R2 | na | 1532 | 0.389 | 0.1554 | Yes |
| 8 | KIR2DL4 | na | 1650 | 0.378 | 0.1727 | Yes |
| 9 | IFNA16 | na | 1944 | 0.354 | 0.1835 | Yes |
| 10 | KIR2DL5A | na | 2285 | 0.328 | 0.1916 | Yes |
| 11 | IFNA6 | na | 2619 | 0.310 | 0.1989 | Yes |
| 12 | PRF1 | na | 3819 | 0.259 | 0.1784 | No |
| 13 | NCR1 | na | 4910 | 0.220 | 0.1589 | No |
| 14 | CD247 | na | 4917 | 0.220 | 0.1707 | No |
| 15 | CHP2 | na | 5296 | 0.206 | 0.1711 | No |
| 16 | BRAF | na | 5517 | 0.199 | 0.1756 | No |
| 17 | ULBP3 | na | 5839 | 0.189 | 0.1766 | No |
| 18 | CSF2 | na | 6383 | 0.171 | 0.1703 | No |
| 19 | KIR3DL1 | na | 6694 | 0.162 | 0.1702 | No |
| 20 | PIK3CD | na | 6778 | 0.159 | 0.1765 | No |
| 21 | RAC3 | na | 7163 | 0.148 | 0.1735 | No |
| 22 | ULBP1 | na | 8418 | 0.118 | 0.1437 | No |
| 23 | BID | na | 8429 | 0.118 | 0.1498 | No |
| 24 | KIR3DL2 | na | 8744 | 0.111 | 0.1468 | No |
| 25 | FASLG | na | 8750 | 0.111 | 0.1527 | No |
| 26 | IFNA7 | na | 9097 | 0.103 | 0.1484 | No |
| 27 | VAV2 | na | 9125 | 0.103 | 0.1532 | No |
| 28 | SOS1 | na | 9467 | 0.096 | 0.1486 | No |
| 29 | ULBP2 | na | 9846 | 0.089 | 0.1425 | No |
| 30 | CASP3 | na | 9912 | 0.088 | 0.1454 | No |
| 31 | RAET1E | na | 10730 | 0.074 | 0.1259 | No |
| 32 | KIR2DS4 | na | 10877 | 0.072 | 0.1256 | No |
| 33 | IFNAR2 | na | 11153 | 0.068 | 0.1213 | No |
| 34 | PIK3CB | na | 11439 | 0.064 | 0.1166 | No |
| 35 | KRAS | na | 11445 | 0.064 | 0.1199 | No |
| 36 | IFNA21 | na | 11545 | 0.062 | 0.1204 | No |
| 37 | NFATC1 | na | 11693 | 0.060 | 0.1195 | No |
| 38 | IFNGR2 | na | 11910 | 0.057 | 0.1163 | No |
| 39 | IFNA2 | na | 12465 | 0.049 | 0.1030 | No |
| 40 | PTPN11 | na | 12805 | 0.045 | 0.0956 | No |
| 41 | IFNB1 | na | 12820 | 0.045 | 0.0977 | No |
| 42 | NFAT5 | na | 12924 | 0.043 | 0.0970 | No |
| 43 | RAC2 | na | 12945 | 0.043 | 0.0988 | No |
| 44 | KIR2DS3 | na | 13074 | 0.041 | 0.0974 | No |
| 45 | MICA | na | 13103 | 0.041 | 0.0988 | No |
| 46 | MAPK1 | na | 13362 | 0.038 | 0.0934 | No |
| 47 | ICAM1 | na | 13556 | 0.036 | 0.0897 | No |
| 48 | TNF | na | 13583 | 0.035 | 0.0909 | No |
| 49 | PLCG1 | na | 13642 | 0.035 | 0.0911 | No |
| 50 | PPP3CB | na | 13986 | 0.031 | 0.0829 | No |
| 51 | PIK3CA | na | 14021 | 0.031 | 0.0836 | No |
| 52 | PTK2B | na | 14053 | 0.030 | 0.0843 | No |
| 53 | MAPK3 | na | 14127 | 0.029 | 0.0838 | No |
| 54 | KLRC3 | na | 15255 | 0.017 | 0.0522 | No |
| 55 | TNFRSF10B | na | 15297 | 0.016 | 0.0518 | No |
| 56 | HLA-E | na | 15330 | 0.016 | 0.0518 | No |
| 57 | HLA-A | na | 15413 | 0.015 | 0.0502 | No |
| 58 | KLRK1 | na | 15632 | 0.013 | 0.0446 | No |
| 59 | HRAS | na | 15878 | 0.010 | 0.0381 | No |
| 60 | PIK3R1 | na | 16145 | 0.007 | 0.0308 | No |
| 61 | MAP2K2 | na | 16236 | 0.006 | 0.0285 | No |
| 62 | TNFRSF10D | na | 16501 | 0.004 | 0.0211 | No |
| 63 | LAT | na | 17047 | -0.002 | 0.0055 | No |
| 64 | FYN | na | 17143 | -0.003 | 0.0029 | No |
| 65 | NRAS | na | 17304 | -0.005 | -0.0015 | No |
| 66 | HLA-B | na | 17543 | -0.007 | -0.0080 | No |
| 67 | RAC1 | na | 18357 | -0.014 | -0.0307 | No |
| 68 | TNFRSF10A | na | 18361 | -0.014 | -0.0300 | No |
| 69 | KIR2DL2 | na | 18402 | -0.014 | -0.0304 | No |
| 70 | HLA-G | na | 18423 | -0.015 | -0.0302 | No |
| 71 | SHC1 | na | 18911 | -0.019 | -0.0432 | No |
| 72 | HLA-C | na | 18927 | -0.019 | -0.0426 | No |
| 73 | KLRC1 | na | 18932 | -0.019 | -0.0416 | No |
| 74 | RAF1 | na | 18969 | -0.020 | -0.0416 | No |
| 75 | ITGAL | na | 19153 | -0.022 | -0.0457 | No |
| 76 | SOS2 | na | 19710 | -0.027 | -0.0603 | No |
| 77 | PPP3R1 | na | 20845 | -0.038 | -0.0910 | No |
| 78 | CHP1 | na | 21142 | -0.042 | -0.0972 | No |
| 79 | SHC4 | na | 21413 | -0.044 | -0.1026 | No |
| 80 | HCST | na | 21923 | -0.050 | -0.1146 | No |
| 81 | GRB2 | na | 22374 | -0.055 | -0.1246 | No |
| 82 | PIK3R2 | na | 22894 | -0.061 | -0.1363 | No |
| 83 | IFNAR1 | na | 22998 | -0.062 | -0.1359 | No |
| 84 | TYROBP | na | 23038 | -0.062 | -0.1336 | No |
| 85 | MAP2K1 | na | 23215 | -0.064 | -0.1352 | No |
| 86 | PAK1 | na | 23429 | -0.067 | -0.1377 | No |
| 87 | GZMB | na | 23756 | -0.072 | -0.1432 | No |
| 88 | PRKCA | na | 23805 | -0.072 | -0.1406 | No |
| 89 | PPP3CA | na | 24064 | -0.076 | -0.1439 | No |
| 90 | NFATC3 | na | 24080 | -0.076 | -0.1402 | No |
| 91 | SYK | na | 24484 | -0.082 | -0.1473 | No |
| 92 | PTPN6 | na | 24509 | -0.083 | -0.1435 | No |
| 93 | VAV3 | na | 24686 | -0.086 | -0.1439 | No |
| 94 | PLCG2 | na | 24806 | -0.087 | -0.1426 | No |
| 95 | IFNGR1 | na | 24941 | -0.089 | -0.1416 | No |
| 96 | TNFSF10 | na | 25116 | -0.092 | -0.1416 | No |
| 97 | ARAF | na | 25299 | -0.095 | -0.1416 | No |
| 98 | ICAM2 | na | 25767 | -0.103 | -0.1495 | No |
| 99 | SH2D1B | na | 26834 | -0.123 | -0.1736 | No |
| 100 | PPP3CC | na | 26944 | -0.124 | -0.1700 | No |
| 101 | FCER1G | na | 27239 | -0.131 | -0.1713 | No |
| 102 | ITGB2 | na | 27251 | -0.131 | -0.1645 | No |
| 103 | MICB | na | 27740 | -0.142 | -0.1708 | No |
| 104 | FAS | na | 28081 | -0.151 | -0.1724 | No |
| 105 | LCP2 | na | 28164 | -0.153 | -0.1664 | No |
| 106 | SH2D1A | na | 28576 | -0.164 | -0.1693 | No |
| 107 | CD244 | na | 28677 | -0.167 | -0.1631 | No |
| 108 | NCR2 | na | 28797 | -0.171 | -0.1572 | No |
| 109 | NFATC4 | na | 29771 | -0.199 | -0.1744 | No |
| 110 | IFNA8 | na | 29826 | -0.201 | -0.1650 | No |
| 111 | SHC3 | na | 30112 | -0.210 | -0.1618 | No |
| 112 | ZAP70 | na | 30212 | -0.213 | -0.1530 | No |
| 113 | PIK3CG | na | 30335 | -0.218 | -0.1446 | No |
| 114 | SHC2 | na | 31857 | -0.283 | -0.1732 | No |
| 115 | IFNA10 | na | 32398 | -0.317 | -0.1715 | No |
| 116 | PIK3R3 | na | 32895 | -0.352 | -0.1666 | No |
| 117 | FCGR3A | na | 32899 | -0.353 | -0.1474 | No |
| 118 | CD48 | na | 32945 | -0.356 | -0.1292 | No |
| 119 | KLRD1 | na | 33201 | -0.376 | -0.1160 | No |
| 120 | IFNG | na | 33359 | -0.391 | -0.0992 | No |
| 121 | TNFRSF10C | na | 33821 | -0.450 | -0.0880 | No |
| 122 | LCK | na | 34075 | -0.497 | -0.0681 | No |
| 123 | VAV1 | na | 34083 | -0.499 | -0.0411 | No |
| 124 | PRKCB | na | 34175 | -0.520 | -0.0153 | No |
| 125 | NCR3 | na | 34338 | -0.572 | 0.0113 | No |
Table: GSEA details [plain text format]

  

Fig 2: KEGG\_NATURAL\_KILLER\_CELL\_MEDIATED\_CYTOTOXICITY      
 Blue-Pink O' Gram in the Space of the Analyzed GeneSet

  

Fig 3: KEGG\_NATURAL\_KILLER\_CELL\_MEDIATED\_CYTOTOXICITY: Random ES distribution      
 Gene set null distribution of ES for **KEGG\_NATURAL\_KILLER\_CELL\_MEDIATED\_CYTOTOXICITY**
